# Effects of a Distributed Form of Constraint-Induced Movement Therapy for Clinical Application: The Keys Treatment Protocol †

**DOI:** 10.3390/brainsci15010087

**Published:** 2025-01-17

**Authors:** Sarah dos Anjos, Mary Bowman, David Morris

**Affiliations:** 1 Department of Occupational Therapy, University of Alabama at Birmingham, Birmingham, AL 35294, USA; mbowman@uab.edu; 2 Department of Physical Therapy, University of Alabama at Birmingham, Birmingham, AL 35294, USA; morrisd@uab.edu

**Keywords:** constraint-induced movement therapy, stroke, rehabilitation, upper extremity

## Abstract

Background/Aim: Currently, there are limited evidence-based protocols for improving upper extremity (UE) motor function after stroke. The Keys protocol, a distributed form of constraint-induced movement therapy (CIMT), delivers CIMT components in fewer hours per day over an extended period, fitting outpatient rehabilitation schedules and third-party payor models. This pilot study aimed to assess the effectiveness of the Keys protocol in enhancing UE capacity and performance poststroke. Methods: Ten adults with chronic stroke (>6 months) participated in an 8-week intervention. The protocol included 22 supervised training sessions (1.5 h each): 4 days/week for 4 weeks, 2 days/week for weeks 5–6, and 1 day/week for weeks 7–8. Participants wore a restraint mitt on the less-affected UE during waking hours and used an adapted transfer package. Outcome measures included the Motor Activity Log (MAL), Wolf Motor Function Test (WMFT), Stroke Impact Scale (SIS), and Zung Depression Scale, assessed pre-treatment, mid-treatment (4 weeks), and posttreatment. Results: Significant improvements were observed in SIS Strength, ADLs/IADLs, Mobility, and Hand Function domains, exceeding MCID thresholds. Memory and Communication domains improved significantly at the 3-month follow-up. WMFT performance times improved, with fewer incomplete tasks. MAL scores for Amount of Use and Quality of Movement increased across all time points. Depressive symptoms significantly decreased posttreatment. Conclusions: The Keys protocol effectively improves UE use, motor function, mood, and quality of life, with the greatest gains observed mid-intervention. These findings support its feasibility and potential for outpatient stroke rehabilitation (ClinicalTrials.gov Registration: NCT05311384).

## 1. Introduction

Stroke is the leading cause of disability in the U.S., resulting in reduced mobility, impaired activities of daily living, social isolation, depression, and unemployment for those with stroke and their caregivers [1]. Consequently, stroke can significantly diminish capacity and performance, two key constructs within the Activities and Participation components of the International Classification of Functioning, Disability, and Health (ICF). Capacity refers to an individual’s ability to do tasks in a controlled, standardized environment (e.g., a laboratory setting), whereas performance reflects what a person can accomplish in real-world contexts [2]. Understanding these constructs is essential for designing effective rehabilitation strategies that address both clinical and everyday challenges.

To combat the high and increasing incidence of stroke, particularly among young adults [3], and the resulting disability coming from stroke worldwide, a few intervention protocols have been developed and recommended by stroke rehabilitation guidelines for improving the affected UE motor function, including constraint-induced movement therapy (CIMT) [4,5]. The signature CIMT protocol systematically delivers intensive treatment sessions in person over 10 consecutive weekdays and behavioral components over one weekend, with a structured approach to maximize outcomes. Each treatment day includes a total of 3.5 h of therapist interaction with 3 h of supervised motor training using shaping and task practice, followed by 30 min dedicated to behavioral strategies known as the Transfer Package, designed to enhance the use of the more-affected upper extremity (UE) in real-life situations. Additionally, the protocol incorporates strategies to encourage consistent use of the more-affected UE, including the restraint of the less-affected UE [6].

Although CIMT is known for its strong evidence, the implementation of the protocol in clinical settings is still challenging and therefore seldom used with all components with fidelity [7]. Multiple barriers for the availability of the CIMT protocol in the clinic have been identified, including its incompatibility with insurance payment policies and the demand for increased therapist time for supervised training, resulting in high costs for implementation [8,9,10,11,12,13,14,15]. Various modified (mCIMT) protocols have been developed in order to overcome these barriers; however, only one or two components of the signature CIMT protocol are usually administered [7,16], with the use of the restraint device and the intensive motor training being the most commonly used in these protocols [16]. The Transfer Package is often omitted, even though it is known to amplify the results of the motor training itself [17,18].

Research suggests that alternative delivery models of CIMT enable the application of all the elements of the CIMT protocol, optimizing therapist time and resources while aligning with the reimbursement policies of many U.S. health insurance providers. Evidence indicates that the original 6 h supervised daily training schedule can be shortened to as little as 2 h/daily without compromising treatment outcomes [19]. Furthermore, additional evidence comes from a study investigating potential changes in the effectiveness of the protocol when adding and omitting elements of the signature protocol. Findings indicated that omitting the transfer package led to a 50% reduction in functional outcomes [18]. This line of evidence was confirmed by brain imaging data that showed reduced brain remodeling in participants with stroke who did not receive the Transfer Package. These results underscore the critical role of the Transfer Package in promoting neuroplasticity and supporting continued movement practice outside the clinical setting, ultimately contributing to more sustainable functional improvements. Therefore, an extended interaction with the Transfer Package might amplify the results observed on the signature protocol.

A distributed form of the signature CIMT, the Keys treatment protocol, consists of all the components of CIMT delivered in fewer hours per day over a greater number of weeks. The name of Keys indicates “Keys to unlock real-world function”. This distributed version of the CIMT treatment protocol is not considered to be a “modified CIMT” because it includes all the components of the signature protocol. Most importantly, in the Keys protocol, the participant interaction with the Transfer Package (TP) is extended to 8 weeks, providing more opportunities for participants to use the affected UE in their own environment. We hypothesize that use of the Keys protocol, including prolonged exposure to the TP, may lead to improvements in UE capacity, performance, and participation that are as significant or more significant than that obtained with the signature CIMT protocol. The purpose of this pilot study was to investigate the effects of the Keys treatment protocol in more-affected UE capacity, performance, and participation and to compare with changes observed with the signature CIMT used in previous studies. This investigation is important because, in contrast to the signature CIMT protocol, the Keys treatment protocol (1) includes an extended interaction with the TP, (2) is more consistent with reimbursement policies in the U.S., and (3) is compatible with implementation in traditional rehabilitation settings.

## 2. Materials and Methods

### 2.1. Study Design

This pilot clinical trial investigated the effect of the Keys treatment protocol on real-world use and motor function of the affected UE of individuals with chronic stroke. Changes in quality of life and occupational performance were also explored in this study. This study was approved by the UAB Institutional Review Board (IRB-300005407), and all participants signed an informed consent.

### 2.2. Participants

In this study, ten individuals were included if they (1) were age 18 years or older; (2) had a stroke at least 6 months prior to enrollment; (3) were able to demonstrate minimum movement criteria of the more-affected UE, including 10 degrees of wrist extension (starting from a fully flexed position), 10 degrees of thumb abduction, and 10 degrees of extension of two additional fingers at all joints; (4) scored less than 2.5 on the Motor Activity Log (MAL); (5) achieved score of 24 or higher on the Mini Mental State Examination (MMSE); (6) demonstrated the ability to comprehend and answer the MAL questions; and (7) had not received a botulin toxin injection or adjustments in anti-spasticity drug regimens within 3 months of treatment. Sensory deficits were not an exclusion. Individuals who were not fluent in English were excluded from the study.

### 2.3. Intervention

**Figure 1:**
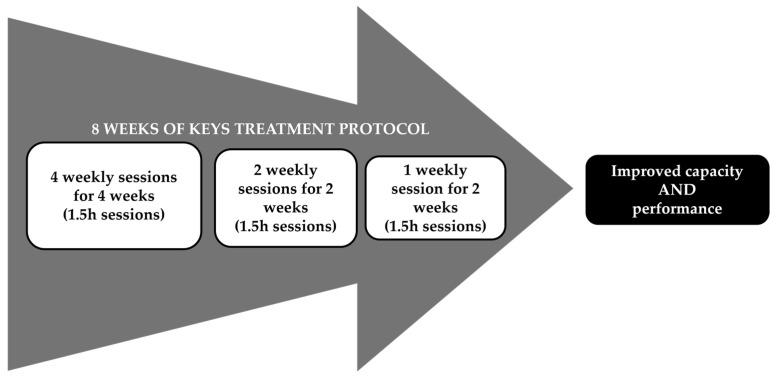
Keys Treatment Protocol Schedule.

All participants received the Keys intervention protocol over an 8-week period with delivery at four times per week for 4 weeks, then tapered to two times a week for 2 weeks, and then one time a week for two weeks (Figure 1). Specific CIMT elements were delivered as previously described [6] except for the following adaptations: (1) 1.5 h session, including supervised movement training carried out for 1 h in the form of shaping [20] and task practice (i.e., 2 shaping tasks), with 30 min allocated for the administration of the Transfer Package [18]; (2) a total of 22 sessions distributed as follows: 4 days/week for the first 4 weeks, 2 days/week for weeks 5 and 6, and 1 day/week for weeks 7 and 8 (Figure 1) [21]; (3) participants used the restraint mitt on their less-affected UE for most of their waking hours for an 8 week period for a target of 90% of waking hours; and (4) interaction with the elements of the Transfer Package throughout the 8 weeks (Table 1). The detailed descriptions and objectives of each element of the treatment protocol as well as the differences between the Keys treatment and the CIMT signature protocol are shown in Table 1.

Another important element of the CIMT protocol is patient education. Although not always explicitly included as part of the treatment, it plays a pivotal role in enhancing patients’ engagement and adherence. Patient education content consists of oral explanations and printed resources that describe how and why CIMT promotes adaptive neuroplasticity, including neuroimagery figures depicting structural gray mater changes following CIMT available in the literature [17]. Information for stroke recovery and health literacy is also covered during educational procedures.

### 2.4. Outcome Measures

The primary outcome measures of this study are the changes in the more-affected UE as it relates to self-reported performance (i.e., real-world use) measured by the [22,23,24], and in motor capacity (i.e., motor function) as assessed by the Wolf Motor Function Test (WMFT) [25,26,27,28]. Secondary outcome measures include the Stroke Impact Scale (SIS) [29,30,31] and the Zung Self-Rating Depression Scale (ZSDS) [32,33]. All measures are reliable and valid to be used with the stroke population.

All measures, except the WMFT, were administered before the treatment (pre-treatment), at the 4-week point in the intervention (during treatment), immediately after the treatment (posttreatment), 1 month after the end of the treatment (follow-up 1), and at 3 months after the end of treatment (follow-up 2). The WMFT was administered by a tester therapist who did not perform the intervention with participants at the pre-treatment, during treatment, and post-treatment time points, and all administrations were recorded and coded. The Functional Ability Scale (FAS) of the WMFT was scored by blinded assessors using videos of the testing sessions. Data were reported in means and changes in scores at pre-treatment, during treatment (after 4 weeks of treatment), post-treatment, and both follow-up assessments.

## 3. Results

**Figure 2:**
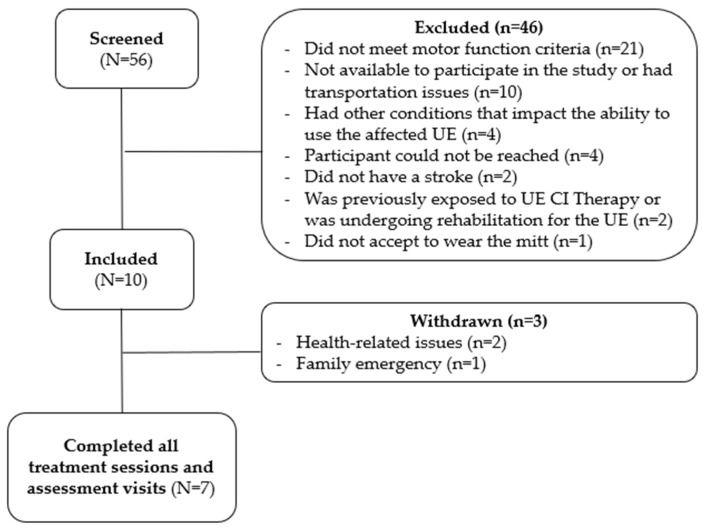
Recruitment flowchart.

Potential participants were recruited from July 2022 to August 2023, and the last participant completed the follow-up assessment visit in January 2024. Overall, 56 individuals were screened, and from those, 46 were excluded (Figure 2). A total of 10 participants were included in this pilot trial; however, three individuals were withdrawn from the study due to reasons unrelated to the treatment protocol. Two participants were withdrawn because of uncontrolled health conditions and one due to family emergency. The seven participants who completed the study attended all treatment sessions and assessment visits.

As shown in Table 2, participants were, on average, 61.7 years old and had a stroke 26.9 months prior to their enrollment in the study. Most participants were White (*n* = 6), had ischemic stroke (*n* = 5), and were right-handed (*n* = 8). Half of the participants had left hemiparesis due to their stroke.

Overall, participants showed improvements in all outcome measures at the 4-week (during treatment) assessment (Table 3). These changes were also observed on the post-treatment assessments, except on the SIS Memory and Communication scores. Although the average of the scores on these two SIS domains was not improved on the post-treatment assessment, the scores had substantially improved on the 3-month follow-up assessment visit.

Participants showed changes in the SIS scores above the minimal clinically important difference (MCID) on the Strength, ADLs and IADLs, Mobility, and Hand Function domains [34]. Substantially improved scores on the SIS were also observed on the Strength and the Mobility domains at the post-treatment and the follow-up assessments. Further notable improvements were observed on the SIS for the ADLs/IADLs and the Hand Function domains during and at the post-treatment assessment and the 3-month follow-up scores. The substantial improvement in depressive symptoms, indicated by the Zung Depression Scale, further underscores the protocol’s benefits.

The only capacity measure administered in this study was the WMFT. The changes observed in the assessment follow the same pattern as the other outcome measures. The difference on the overall performance time from the pre-treatment assessment was higher than the MCID [27] at both during (−1.5 s) and post-treatment (−2.09 s) time points. Quality of movement during the performance of the WMFT tasks was assessed using the Functional Ability Scale. As seen in Table 3, there was no change in the scores obtained on this scale, but the number of tasks that the participants were unable to complete decreased during and after the treatment.

**Figure 3:**
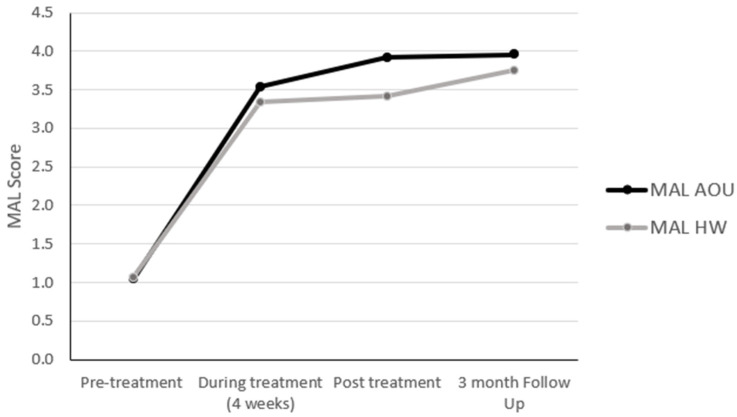
Changes in the MAL scores.

Participants showed improvement in the use of the more-affected upper extremity assessed by the MAL in both the amount of use and quality of movement (Figure 3). The difference in the scores obtained at the pre- and during treatment time points (4-week assessment visit—AOU: 2.5, and QOM: 2.2) were, on average, higher than the 0.5-point MCID previously reported in the literature [23,24]. The same was observed at both the post-treatment time point and 3-month follow-up visit where the differences in both scales of the MAL (AOU: 2.9 and QOM: 3.0; AOU: 2.3 and QOM: 2.6, respectively) were higher than the MCID previously reported.

Of the ten participants initially included in the trial, seven completed the treatment. Three participants withdrew due to reasons unrelated to the clinical trial. All participants who completed the trial had 100% attendance in both treatment and assessment visits. No adverse events or falls were reported.

## 4. Discussion

This study protocol delivered all the elements of the signature CIMT protocol [4] in a distributed schedule, in shorter supervised sessions, to make it potentially reimbursable and time-efficient. Specifically, adjustments were made to the treatment duration, session length, and weekly frequency. The purpose of this pilot trial was to preliminarily assess the effectiveness of a distributed CIMT, i.e., the Keys treatment protocol, addressing barriers that limit access to traditional CIMT in clinical practice. Although the Keys protocol retained all the core elements of CIMT, adaptations were made to the session duration and frequency, with use of the mitt 90% of the waking hours daily throughout the 8 weeks of treatment. These changes aimed to address common barriers to implementing traditional CIMT, such as the time-intensive nature of the intervention for the therapist, patient adherence to wearing the mitt, and consistent engagement in transfer package activities.

This pilot trial evaluated the impact of the Keys protocol on motor capacity, upper extremity (UE) use, depression, activities of daily living (ADL), and overall quality of life in individuals with chronic stroke. Notably, participants showed improvements in the Hand Function domain of the SIS, surpassing results reported in the literature protocol [35]. Important changes in UE use and quality of life were observed during and after treatment, with these improvements maintained three months after the intervention. The results indicate that the distributed model may enhance outcomes, as evidenced by superior improvements in the MAL measures compared to previous studies [18,35,36,37,38]. Importantly, the positive effects persisted or were even amplified at follow-up. A substantial improvement on performance time measures by the WMFT was observed; however, this change was not reflected in the Functional Ability Score of the WMFT.

The variability in CIMT protocols across the literature makes direct comparisons challenging [16]. One critical element often neglected in other protocols is the behavioral strategy component known as the “Transfer Package”. In the Keys protocol, participants engaged with these strategies for eight weeks, compared to the typical two-week period in the signature CIMT [6]. The extended exposure to the Transfer Package likely contributed to sustained improvements in UE use, reinforcing motor gains through continuous practice in real-world contexts.

The Transfer Package is essential for integrating the more-affected limb into daily activities, promoting motor retention by encouraging functional use outside therapy. This component involves problem-solving tasks, home-based practice, and self-monitoring [18]. Research indicates that including the Transfer Package in CIMT significantly enhances long-term motor outcomes, helping patients maintain improvements and avoid reverting to compensatory behaviors [18,39,40,41].

Despite strong evidence supporting CIMT and its endorsement in stroke rehabilitation guidelines [4,5], real-world implementation remains challenging. Barriers include the higher number of hours required from patients and clinicians to engage in the supervised sessions, maintaining patient adherence, the logistical administration of Transfer Package elements, lack of reimbursement from third-party payers, and lack of therapists training on the application of CIMT [8,9,10,11,12,13,14,15,42]. These challenges highlight the need for innovative adaptations of CIMT to align with the current healthcare scenario [43]. The Keys treatment protocol addresses some of these issues, and more efforts to reduce barriers to delivering this evidence-based intervention should be considered. Additionally, the Keys protocol presents a feasible and potentially reimbursable delivery option for implementing CIMT in the clinical settings while achieving outcomes comparable to the signature protocol.

### 4.1. Limitations

The primary limitation of this study is the small sample size, which restricts the ability to generalize the findings to a broader population. Furthermore, with a small sample, individual variability among participants can skew the findings and limit their reliability. Although the incidence of stroke has increased [44,45,46], there are multiple barriers to recruitment, including challenges caused by the COVID-19 pandemic, transportation limitations, and difficulties in controlling health conditions (e.g., arterial hypertension).

While the results are promising, larger studies with comparison groups are required to better understand the potential of the Keys treatment protocol and identify what results are associated specifically with this protocol. Although the MAL has shown a high correlation with objective measures (e.g., accelerometry and neuroimaging data) [17,23], another potential limitation is the use of self-reported (perceived) performance outcomes, as participants may provide inaccurate information due to memory issues or personal biases. Despite these limitations, the MAL is an appropriate outcome measure for this study because of its strong psychometric properties, and it has been used extensively in CIMT and other stroke rehabilitation studies. Also, even with recent technological advances, there are still challenges in using activity monitors with the specificity needed to accurately measure actual performance improvements.

### 4.2. Future Directions

To overcome the logistical challenges and barriers posed by signature CIMT, future research should explore the feasibility of delivering adapted protocols remotely. Telehealth approaches could increase access to CIMT, especially in rural or underserved areas, and reduce the burden of frequent clinic visits. Since the finding of this study showed such robust changes after just 4 weeks of treatment, the intervention could be studied for 4 weeks of treatment. Further investigation of the effectiveness of the Keys treatment protocol should include larger sample sizes as well as comparison groups such as the signature CIMT protocol and interventions commonly delivered in the clinical settings. Additionally, future studies could investigate changes in motor performance using wearable sensors and accelerometers to provide objective measures of improvement [47,48,49,50]. These technologies can capture real-time data on movement patterns and intensity, offering deeper insights into patients’ progress both during and after treatment.

## 5. Conclusions

The results of this study highlight the positive impact of the adapted Keys treatment protocol on motor capacity, upper extremity performance, ADLs, and overall well-being in individuals with chronic stroke. Significant improvements were observed across multiple domains of the SIS, including Hand Function, Mood, Mobility, and Participation, with most gains maintained or amplified at the three-month post-treatment assessment.

This distributed version of CIMT is promising for addressing barriers associated with reimbursement and therapist scheduling in rehabilitation settings by distributing the treatment delivery and applying behavioral strategies over a longer period. While the small sample size limits the generalizability of these findings, the results point to the potential for this approach to enhance functional recovery and quality of life. Future research should focus on larger trials, potentially integrating telehealth solutions, reducing the overall number sessions, and utilizing objective measures (e.g., wearable sensors) to validate these findings and explore sustainable delivery models for CIMT in clinical practice.

## Figures and Tables

**Table 1:** Parameters and Components of the CIMT and their Application in the Signature and the Keys Treatment Protocols.

CIMT Components	Signature CIMT	Keys Protocol	Purpose/Intent
*Parameters of the Intervention Protocols*			
Frequency and duration of in-clinic sessions	3.5 h daily sessions for 10 consecutive weekdays (total of 35 h)	1.5 h sessions distributed in 8 weeks: four sessions weekly for 4 weeks, two sessions weekly for 2 weeks, and one session weekly for 2 weeks (total of 33 h)	All components of CIMT are delivered throughout the treatment period. The Keys treatment protocol allows patients to interact with the protocol components over an extended period in a distributed format. This approach maintains a similar total number of hours as the signature protocol. The treatment includes strategies to enhance self-efficacy, patient education, and structured interactions, providing multiple problem-solving opportunities for both therapist and patient.
Overall engagement duration (therapist/patient)	6 weeks: 2 weeks of in-clinic sessions and four weekly follow-up calls for MAL administration	12 weeks: 8 weeks of in-clinic sessions, and four weekly follow-up calls for MAL administration	The engagement with the components of the CIMT protocol includes supervised training, tasks performed at home, and interaction with the Transfer Package not only during the treatment days but also during follow-up throughout the first month after the end of the treatment sessions.
*Component 1: Movement Training*			
Shaping	TD1–TD10	TD1–TD22	Shaping in CIMT is a systematic approach to movement training with repeated timed trials in which progress is made in small steps. Primary purpose is to build confidence; Safety emphasized throughout; Use of four strategies of modeling, coaching, feedback, and encouragement; Just-right challenge with UE skilled tasks performed typically between 30–45 s trials; Coaching and feedback for providing knowledge of results emphasized throughout; Speed and quality of movement training addressed on each trial.
Task practice	TD1–TD10	TD1–TD22	Task practice is movement training that focuses on continuous movement throughout the 10–30 min functional activity. Tasks are usually functional in nature; Safety emphasized throughout; Modeling, coaching, feedback, and encouragement are used; Feedback in summative and not on a trial-by-trial basis; Often emphasizes activities that are both meaningful and appropriately challenging for the individual.
*Component 2: Transfer Package*			
Behavioral contract	TD1 and TD6	TD1; reviewed as needed	An agreement between the patient, caregiver, and therapist that addresses mitt use and more-affected UE use within the patient’s current routine. Safety emphasized as first consideration and addressed throughout the document; the last category discusses when the mitt is removed and when the less-affected hand is used due to safety reasons for tasks like handling hot liquids/ foods, ascending and descending stairs, walking with a cane, driving a car, etc. Caregiver support defined; Adaptive equipment and adaptive strategies initiated for ADL; Increases accountability for use as part of therapeutic alliance.
Daily schedule	TD1–TD10	TD1–TD22	A document that serves as an account of daily activities in the clinic setting by the therapist. Details mitt use in the clinical setting; The therapist documents mitt use and activities performed in the clinic setting and mirrors the documentation of the mitt use outside of the clinic setting in the home diary; Fosters adherence and accountability.
Home diary	TD1–TD10, including weekends	TD1–TD 22, including weekends	A daily diary kept by the patient or caregiver detailing mitt usage outside of the clinic setting. Safety emphasized with patient’s account of mitt use and activities performed; Provides information about the number of waking hours; Increases accountability for use of the more-affected UE and the mitt as part of therapeutic alliance; Reviewed daily with patient and caregiver if present.
Home skill assignment (HSA)	TD2–TD10, including weekends	TD2–TD22, including weekends	A set of 10 activities is selected daily in a process with the patient and therapist (and caregiver if present) to be performed outside of the clinic setting, with five easier activities and five more challenging activities selected. Emphasis on patient’s generalized use of the more-affected UE by performing activities they may not have performed since the stroke, such as bathing the body, flushing the toilet, etc.; Safety is emphasized; Adaptive strategies and adaptive equipment employed as needed.
Daily administration of the HW Scale of the MAL	TD1–TD10 30 items of HW Scale on TD1 and TD6 and ½ of MAL HW on the remaining TDs.	TD1–TD22 ½ of MAL HW Scale each TD	Administration of MAL HW Scale daily to track progress through treatment. Fosters problem solving; Tracks progress of the more-affected UE through treatment; Safety emphasized; Adaptive equipment discussed and trialed as needed.
Problem solving	TD1–TD10	TD1–TD22	A process of finding ways to use of the more-affected UE better in tasks the patient already performs and for new tasks the patient is trying. Safety emphasized; Promotes effective carry over of more-affected UE capacity gains into daily performance; Discussion of the use of adaptive equipment or adaptive strategy development as needed.
Home practice after treatment	Developed over treatment and trained on the TD9 and TD10, to be carried out indefinitely	Developed over the treatment and trained on TD22, to be carried out indefinitely	A document-based home skill assignment that outlines use of the more-affected UE in daily life for eight activities and practicing skills for two activities, with instructions provided for use of the more-affected UE, addressing safe continued progress. Safety emphasized; Effective carry over of more-affected UE capacity gains into daily performance; Adaptive equipment and adaptive strategies emphasized as needed.
Weekly administration of the MAL	Weekly for 4 weeks after discharge	Weekly for 4 weeks after discharge	Administration of the MAL by telephone or video call to monitor progress with the use of the more-affected UE. Fosters problem solving; Tracks progress of the more-affected UE for the first 4 weeks after treatment; Safety emphasized.
*Component 3: Constraint (Encourage Use) of the More-Affected UE*			
Mitt use on less-affected UE	TD1–TD10 for 90% of waking hours	TD1–TD22 for 90% of waking hours	A mitt restraint is worn on the less-affected hand for 90% of waking hours to encourage use of the more-affected UE. Safety emphasized regarding mitt use in the behavioral contract, home diary, daily schedule, HSA, shaping, and task practice; Mitt is removed for any task that involves water; Mitt is removed if a device is used for ambulation; Mitt is not worn during sleep and naps; Mitt is on weekdays and weekends.
Any other methods for reminding the patient to use the more-affected UE	TD1–TD10	TD1–TD22	The use of reminders, apps, and any visual aids to encourage the use of the more-affected UE. Doorknob hangers; Sticky notes/bright stickers; Reminder apps; Wearable sensors.

CIMT = Constraint-Induced Movement Therapy; TD = treatment day; ADL = activities of daily living; UE = upper extremity; MAL = Motor Activity Log; HW = How Well scale of the MAL.

**Table 2:** Demographic characteristics of the participants.

Participant	Sex	Age (y)	Ethnicity/Race	Type of Stroke	Time Since Onset (m)	Affected Side	Pre-Morbid Handedness
1001	M	49	AA	I	20	R	R
1002	M	64	W	I	19	R	R
1003	F	46	AA	I	20	R	R
1004	F	71	W	H	50	R	R
1005	F	65	W	I	11	L	R
1006	F	58	AA	U	56	L	R
1007	M	61	W	U	24	L	R
1008	M	72	W	I	9	R	L
1009	M	67	AA	U	54	L	L

M = male; F = female; AA = African American or Black; W = White; I = ischemic; H = hemorrhagic; U = unsure; m = months; R = right; L = left.

**Table 3:** Average scores on outcome measures on each time point.

Outcome Measures	Overall Scores				Difference in Scores Since Pre-Treatment		
	Pre-Treatment	During Treatment (4 Weeks)	Posttreatment (8 Weeks)	3-Month Follow-Up	Difference 4 Weeks–Pre	Difference Post–Pre	Difference 3-Month Follow-Up–Pre
MAL AOU	1	3.5	3.9	4	2.5 *	2.9 *	3 *
MAL QOM	1.1	3.3	3.4	3.7	2.2 *	2.3 *	2.6 *
WMFT—Median Performance Time (s)	5.32	3.82	3.23	.	−1.5 *	−2.09 *	.
WMFT—Median Functional Ability score	3	3	3	.	0	0	.
WMFT—Number of Non-Completed Tasks	3	1	2	.	−2	−1	.
SIS—Strength	51.8	59.8	65.2	65.2	8	13.4 *	13.4 *
SIS—Memory	59.8	63.9	61.6	67.7	4.1	1.8	7.9
SIS—Mood	64.2	63.9	65.5	69	−0.3	1.3	4.8
SIS—Communication	90.5	91.8	90.3	93.4	1.3	−0.2	2.9
SIS—ADLs/IADLs	56.8	70.4	77.1	81.1	13.6 *	20.3 *	24.3 *
SIS—Mobility	77	79.4	82.9	84.1	2.4	5.9 *	7.1 *
SIS—Hand Function	36.1	60.7	65	70.7	24.6 *	28.9 *	34.6 *
SIS—Participation	56.4	69.6	75	76.8	13.2	18.6	20.4
SIS—Recovery	58.9	59.3	70	76.4	0.4	11.1	17.5
ZDS	34.1	32.1	30.4	31	−2	−3.7	−3.1

MAL = Motor Activity Log; AOU = amount of use; QOM: quality of movement; WMFT = Wolf Motor Function Test; SIS = Stroke Impact Scale; ZDS = Zung Depression Scale. * Differences above the minimal clinically important difference (MCID) for that assessment.

## Data Availability

Due to Institutional restrictions, results will be available at https://clinicaltrials.gov/study/NCT05311384. For additional information or inquiries, please contact the corresponding author.
